# Insulitis in human type 1 diabetes: lessons from an enigmatic lesion

**DOI:** 10.1093/ejendo/lvae002

**Published:** 2024-01-17

**Authors:** Noel G Morgan

**Affiliations:** Department of Clinical and Biomedical Science, Islet Biology Exeter (IBEx), Exeter Centre of Excellence in Diabetes (EXCEED), University of Exeter Medical School, Exeter EX2 5DW, United Kingdom

**Keywords:** type 1 diabetes, islets of Langerhans, autoimmunity, endotype

## Abstract

Type 1 diabetes is caused by a deficiency of insulin secretion which has been considered traditionally as the outcome of a precipitous decline in the viability of β-cells in the islets of Langerhans, brought about by autoimmune-mediated attack. Consistent with this, various classes of lymphocyte, as well as cells of the innate immune system have been found in association with islets during disease progression. However, analysis of human pancreas from subjects with type 1 diabetes has revealed that insulitis is often less intense than in equivalent animal models of the disease and can affect many fewer islets than expected, at disease onset. This is especially true in subjects developing type 1 diabetes in, or beyond, their teenage years. Such studies imply that both the phenotype and the number of immune cells present within insulitic lesions can vary among individuals in an age-dependent manner. Additionally, the influent lymphocytes are often mainly arrayed peripherally around islets rather than gaining direct access to the endocrine cell core. Thus, insulitis remains an enigmatic phenomenon in human pancreas and this review seeks to explore the current understanding of its likely role in the progression of type 1 diabetes.

**Significance**

Type 1 diabetes is widely believed to be caused by a direct loss of pancreatic β-cells mediated by an immune cell assault. In support of this, immune cells are known to infiltrate the pancreas in type 1 diabetes in a process known as insulitis. However, studies of insulitis in humans with recent-onset type 1 diabetes have revealed that the extent of insulitis is often rather modest and can vary according to the age at onset of the individual. Thus, the current model of immune-mediated destruction may be an over-simplification and this review summarises the present state of our understanding. It also highlights areas where more information is required to provide an improved framework for the design of targeted interventions.

## Introduction

The term “diabetes” refers to a series of disorders associated with the production of unusually large volumes of urine which, at least according to archaic methods of diagnosis (tasting!) may be either sweet (termed “mellitus” from the Greek for honeyed) or insipid.^1^ The sweetness of the urine derives from the presence of glucose and this feature provides for the current clinical definition since when the capacity for glucose reabsorption by the kidney is exceeded (typically when blood glucose levels reach ∼10 mmol/L), the hexose is not recovered from the urine in sufficient quantity, and the body loses a key source of energy production by excretion. This situation develops when secretion of the polypeptide hormone, insulin, is deficient and/or tissues become insensitive to insulin such that their capacity to accumulate glucose from the extracellular fluids is impaired. As such, diabetes is caused by relative or absolute insulin deficiency and is perceived as a condition of starvation since circulating glucose is not utilised for tissue energy production.

Differences in the manifestation and clinical course of diabetes across the lifespan fuelled the concept that diabetes is not a single disease and led to the recognition of two principal subtypes; an early-onset (“juvenile”) form of the disease and an older onset (“maturity”) form.^2^ These definitions are now known to be too arbitrary (not least because either form can occur throughout the life course) and the subsequent emergence of designations recognising “type 1” (formerly “juvenile”) and “type 2” diabetes has been accepted. Other diabetes variants have also been defined which include familial conditions associated with specific genetic mutations,^3,4^ as well as forms of gestational diabetes arising during pregnancy.^5^

Type 2 diabetes presents the largest social and economic burden, since it affects hundreds of millions of individuals across the world and is increasing at an alarming rate, globally.^6,7^ Epidemiological and demographic analyses mean that type 2 diabetes is understood primarily as a condition associated with aging and changing lifestyle choices, although, sadly, it is increasingly diagnosed in adolescents and children.^8,9^ There may also be phenotypic differences among subjects with type 2 diabetes since the symptoms can be manifested in those who are lean as well as among those who are overweight. In the former case, the underlying basis may correlate more readily with β-cell dysfunction than with declining insulin sensitivity, which is accelerated in obesity. By contrast, type 1 diabetes is often more acute in onset and associated with intense metabolic changes. It occurs frequently in children (but can be manifested at any age^10,11^) and displays features of autoimmunity which often appear unexpectedly and lead to the profound loss of circulating insulin. Thus, the progression of types 1 and 2 diabetes are markedly different and, although attempts have been made to offer harmonising pathophysiological mechanisms,^12^ the weight of evidence implies a categorically distinct underlying pathophysiology in each case.

## Immunopathology of the human pancreas in type 1 diabetes

Against this background, the present review seeks to assist the reader in gaining an appreciation of the current understanding of the pathophysiology of human type 1 diabetes and, to achieve this, it focusses on one principal physiological location: the islets of Langerhans in the pancreas. This is because type 1 diabetes is essentially a pancreatic condition despite the fact that there are a multitude of associated features that can be detected peripherally (including alterations in various immune cell phenotypes, generation of islet autoantibodies, secretion of cytokines, chemokines, etc.).^13–17^ Having made this point, a dilemma emerges because the pancreas is an inaccessible gland physiologically and it is not open to high-resolution scrutiny in living individuals by any readily available, ethically acceptable, method. Hence, unlike the situation for many other diseases, the understanding of human type 1 diabetes has been derived largely by inference rather than from direct observation of the disease process *in situ*. This yawning gap in understanding has been increasingly recognised as inhibitory to progress in the development of disease-modifying therapies and is now being addressed. However, the goal is not trivial and relies heavily on the study of pancreas tissue obtained post-mortem from individuals diagnosed with type 1 diabetes. In one important Norwegian study,^18^ pancreas biopsy samples were recovered from living subjects newly diagnosed with type 1 diabetes in adulthood (with ethical permission) but only 6 of an intended 60 such samples had been harvested when the sampling was halted due to surgical complications.^18^ Thus, aside from these few (very informative) samples, our window into the pancreas in type 1 diabetes is mainly restricted to the gland at terminal demise. This then raises further issues since many of the extant samples come from subjects with relatively long-standing disease. Moreover, for those rarer cases with more recent-onset diabetes, death was often associated with severe metabolic derangement (including ketoacidosis) and/or it followed periods of intervention in intensive care facilities. Such conditions may influence the status of the pancreas at the point of harvest,^19^ meaning that uniquely disease-associated changes might be obfuscated.

In addition to the Norwegian “DiViD” biopsy samples noted above, the largest pancreas biobanks which, together, have yielded much of the current understanding are located in the UK and USA and some relevant images can be viewed at https://www.pancreatlas.org. However, the first such collection (which was ground-breaking in terms of vision and insight) was compiled by Gepts^20^ in the 1960s and is held in Belgium. The most contemporary and largest collection of pancreas samples from people with type 1 diabetes is held under the auspices of the Network for Pancreatic Organ Donors (nPOD) in Florida^21,22^ who remain active in both the compilation and analysis of their samples using increasingly sophisticated methodologies. Network for Pancreatic Organ Donors has consistently championed a “team science” approach in which investigators drawn from across the globe have formed networks bringing complementary expertise to help unravel the mysteries of the disease. The results have been truly enlightening^22^ but the collection has restrictions since it contains relatively few recent-onset cases from young children. As a result, many of the outputs reflect the status of the disease in people who were in adolescence or beyond at onset and/or with longer duration disease. By contrast, the UK collection (compiled originally by Foulis^23^ in the 1980s from historical autopsy specimens and now held as the “Exeter Archival Diabetes Biobank”, EADB) contains a larger number of cases from young children who died soon after disease onset. Indeed, most of the samples held in EADB come from people under the age of 20 years at the time of death.^24^ Thus, these two larger biobanks are truly complementary. Nevertheless, it is important to mention that the nPOD biobank has a further notable advantage, in that a more complete clinical history of each of donor is available. Accordingly, this allows correlation of any observed pancreatic pathologies with other clinical parameters, including factors such as HLA haplotype and autoantibody status. Such data are largely missing for the samples held in the EADB biobank (because of its historical origins) and this can be a limitation when comparisons are made among samples from different subjects.

## The phenomenon of insulitis

Using both EADB and nPOD tissues, much work has focussed on the process of insulitis which was initially recognised as a feature of type 1 diabetes in animal models of the disease such as the non-obese diabetic (NOD) mouse^25^ and BioBreeding rat.^26^ It has become apparent, however, that the disease in humans displays rather different features from some of those evident in the animal models^27^ where insulitis was first characterised. Insulitis is a process of inflammation in which cells of the immune system invade the pancreas and accumulate in proximity to, and within, pancreatic islets of Langerhans. In rodents, this process occurs increasingly intensively as the disease progresses until most islets display extensive infiltration. This observation has fuelled the concept that, in type 1 diabetes, β-cell death is mediated by an immune mechanism involving large-scale infiltration of cytotoxic immune cells (principally CD8+ T-cells) into the islets.^28,29^ However, this feature of intense insulitis is largely absent when human pancreases from people with type 1 diabetes are examined. One possible explanation might be that the immune cell infiltration occurs earlier in the disease process in humans and that it has subsided by the time of pancreas harvest after disease onset. This seems unlikely, however, since analysis of samples from people who had died prior to clinical onset but who were judged to be progressing to clinical disease, failed to reveal evidence of intense insulitis. Indeed, the evidence in human pancreas was so limited that insulitis was dubbed an “elusive lesion” in a seminal review by In’t Veld.^30^ This, then, raises questions as to whether insulitis is truly a characteristic feature of human type 1 diabetes and if it could be causative for disease.

**Figure 1.**
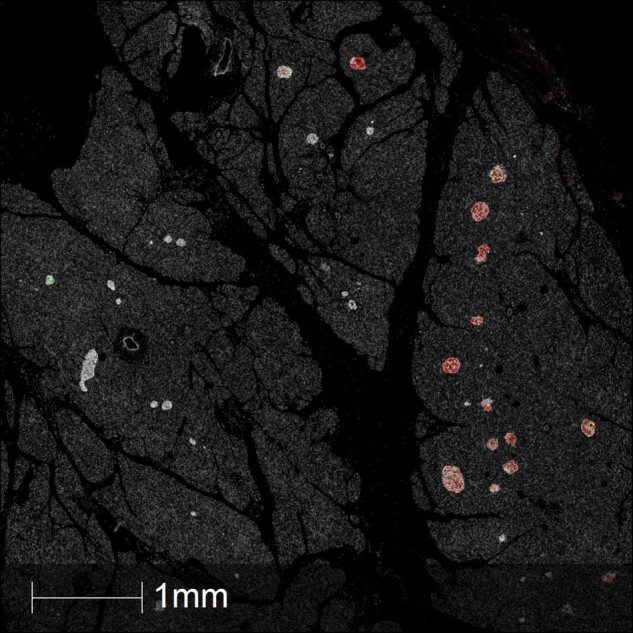
The lobularity of β-cell destruction in human pancreas in type 1 diabetes. Low power view of a section of human pancreas recovered from an individual with recent-onset type 1 diabetes. The section contains three lobes (lower left, upper middle, and lower right) bearing multiple islets each having a differential extent of β-cell loss. Islet hormones were detected by immunofluorescence to reveal insulin (red), glucagon (white), and somatostatin (green). The lobe to the lower left contains islets which are devoid of insulin, that in the upper middle has islets with a varying extent of insulin immunostaining, while the islets in the lobe to the lower right appear insulin replete. Image kindly provided by Dr Pia Leete.

The answers to these questions remain a matter of debate, although dogma still favours the notion that insulitis and CD8+ T-cell-mediated cytotoxicity underlie β-cell loss in human type 1 diabetes. One possibility that might account for its elusive detection is that insulitis could occur in a non-uniform manner across the pancreas such that some regions are affected more readily than others and that these are sampled differentially during sectioning of fixed pancreatic tissue for analysis. Credence has been lent to this idea by the finding that β-cell loss displays regional differences and that some areas of the pancreas contain islets in which β-cell loss is extensive, whereas islets in other regions of the same gland appear normal and retain β-cells in greater numbers (Figure 1). This has been referred to as “lobularity” of progression^31,32^ since islets of each type tend to be found in groups, often aligned within a single lobe of the gland. However, it must also be noted that these differences are not always restricted to groups of islets but can sometimes be seen even among individual islets lying in close proximity. Overall, it can be concluded that neither the timing of pancreas harvest nor the differential sampling of specific pancreatic lobes appears to account for the elusivity of insulitis. Rather, we are left with the possibility that insulitis is less intense and much less prevalent among human islets in type 1 diabetes than in those of the most-studied animal models.

In considering these arguments, the tacit conclusion might be drawn that “insulitis” is a readily identifiable process with a firm and widely accepted definition; but this is not the case. We had previously proposed a criterion of five CD45+ cells (lymphocytes) per islet to define insulitis.^33^ This was based on studies of the islets of children without diabetes control where fewer than this number of lymphocytes were found routinely. However, in order to consolidate opinion, an international consensus group was formed to propose a working definition that would be accepted universally.^34^ What emerged is, on the one hand, useful while, on the other, it serves to emphasise that the process remains enigmatic. Thus, it was proposed that insulitis should be confirmed where as few as 3 islets, each with at least 15 associated CD45+ cells, could be found in a pancreas. Given that a human pancreas might typically contain ∼1 million islets, the notion that 3 inflamed islets would constitute a positive outcome provides an insight into the dimensions of problem. Very recently, proposals have been advanced that this definition should be revised in a manner which accounts for the increasing capacity to analyse pancreas images containing large numbers of islets using software-based methods (rather than “by eye” as in earlier studies).^35^ This has spawned the proposal for a “30–30” rule whereby the presence of ≥30 CD3+ (T-) cells per mm^2^ among a total of 30 islets tissue would constitute a definition of insulitis. In reflecting on the merits of this, it must be emphasised that the measurement of CD3+ cell infiltration as a criterion may not prove ideal because islets can be infiltrated by lymphocytes that lack CD3 and it will be necessary to ensure that all counted lymphocytes are genuinely islet-associated by careful evaluation of the region considered as influential to each islet. Moreover, contrary to accepted wisdom, another study has argued that the absolute density of immune cells is typically greater in the exocrine compartment of the pancreas than in the islets, in type 1 diabetes.^36^ Thus, the debate about how best to define insulitis rumbles on and its final resolution is of more than academic value. Rather, it is critical to a complete understanding of disease aetiology and to the design of improved therapeutic reagents.

## Insulitis profiles at increasing ages at diagnosis of type 1 diabetes

**Figure 2.**
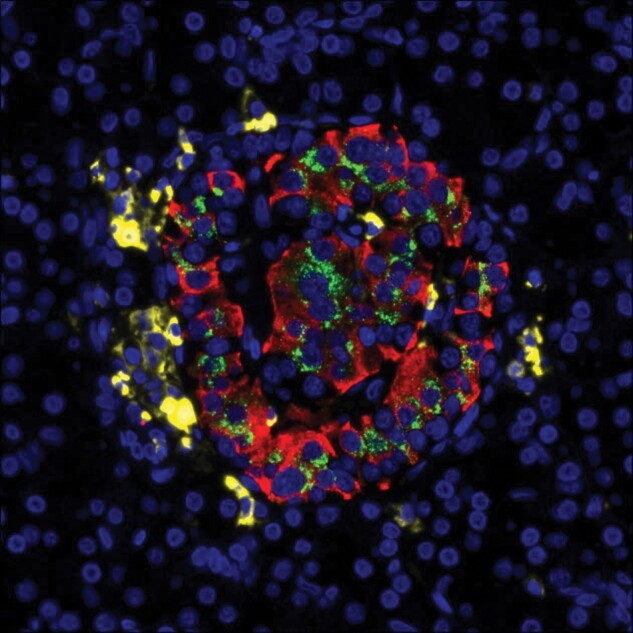
Arrangement of lymphocytes in an insulitic lesion associated with a single islet of Langerhans from a subject with recent-onset type 1 diabetes. High power image showing a single islet of Langerhans from an individual with type 1 diabetes to illustrate the distribution of proinsulin (green), insulin (red), and infiltrating lymphocytes (CD45+; yellow). The islet retains residual insulin-immunopositive β-cells and may be under attack from CD45+ lymphocytes. However, these are mainly arranged around the periphery of the islet with very few lymphocytes detected in close physical contact with β-cells within the islet core. Image kindly provided by Dr Pia Leete.

Analysis of the pancreases of people of different ages at diagnosis has highlighted the importance of a robust definition of insulitis since it has been established that the extent and frequency of immune cell infiltration varies according to age in children and young people with type 1 diabetes.^37,38^ In particular, early work revealed that islet immune cell infiltration can be detected in very young children to a much greater extent than in those who are older.^37,38^ Moreover, the pattern and extent of this process varies according to the proportion of β-cells retained in insulin-containing islets in a manner which suggests dynamic temporal regulation.^33^ As such, immune cells appear to initially reach the islets in quite large numbers when β-cells are present in abundance but then migrate away as β-cell numbers decline. Thus, it can be concluded that (almost irrespective of the precise quantitative definition applied) β-cell loss is accompanied by insulitis in the youngest children; noting however, that, even in these subjects, the extent of infiltration rarely achieves levels equivalent to those seen in NOD mice. By contrast, among older children (those into their teenage years at diagnosis) there is much less evidence of active insulitis at onset^37^ and the idea that their β-cells are destroyed by an abundant influx of CD8+ T-cells is based more on comparative inference than on firmly documented evidence. It is also the case that immune cells are found most often at the periphery of islets in human type 1 diabetes (Figure 2), whereas they penetrate the endocrine cell core more rarely so that very few lie in direct contact with β-cells. This implies that the killing mechanism may operate remotely (eg, by release of cytokines rather than via direct cell contact) and/or that the process is highly inefficient, at least in those diagnosed as teenagers or beyond. Following on from these observations, it must then be questioned whether the underlying causative processes are similar in all children or if these differ according to age. Suggestions that the latter may be true are now beginning to achieve traction.

This notion was mooted following a detailed analysis of the composition of the immune infiltrates associated with islets in either young children (<7y) or those diagnosed at older ages (>13y) which revealed differences in immune cell composition.^37^ In the youngest children studied, these infiltrates contained predominantly CD8+ T-cells but they were accompanied by almost equal numbers of a second type of lymphocyte defined by the presence of CD20. Such cells are typically considered as B-lymphocytes and, in this regard, the insulitis in young children displays features similar to those seen in NOD mice, where immune cell infiltration is more intense and T-cells and B-cells are abundant.^38^ The majority of B-cells seen in human insulitis lack CD138,^33^ a typical marker of antibody-secreting B-cells, suggesting that they fulfil a role which is unrelated to antibody secretion. One particularly intriguing phenomenon is that the disposition of the CD20 protein on the cell surface appears to change according to the proximity of the cells to islets with evidence of increasing surface aggregation as the B-cells migrate close to islets.^39^ They also appear to interact directly with CD8+ T-cells in the islet vicinity leading to the proposition that the influent B-cells may be involved in antigen presentation to CD8+ T-cells close to the islet site.^39^ This hypothesis remains to be proven but offers a potential mechanism by which T-cell activation might be enhanced close to the site at which β-cell death occurs. Overall, such evidence would be consistent with a process in which activated CD8+ T-cells are engaged to encounter β-cells directly in the islets of young children and that the principal mode of killing involves a contact-mediated cytotoxicity.

When turning to older children and adolescents, it is clear that the complement of CD20+ cells in the insulitis is much reduced by comparison with that seen in young children. Indeed, the total number of all immune cells is much lower on average and, as noted above, few CD8+ cells penetrate into the islet core. Perhaps, then, the mechanisms by which insulin is depleted differ in these subjects by comparison with those who are younger at onset? In support of this, it was also noted in these cases, that many islets retain at least some residual β-cells at the time of diabetes onset^40,41^ and that the proportion of insulin-positive islets showing evidence of even mild inflammation is low (often fewer than 30%).^37^ Thus, in these older children, type 1 diabetes is manifested symptomatically when significant numbers of β-cells are still present and the extent of insulitis is modest. In this situation, it is feasible that a defect in insulin secretion may contribute significantly to the reduction in circulating insulin concentrations^42^ rather than this being caused solely by the annihilation of β-cells, as in younger children. This does not mean that β-cell demise is not a feature of older onset type 1 diabetes because these cells are undoubtedly lost (especially with increasing disease duration).^41^ However, the rate and extent of their loss appears markedly different between the two groups of individuals and this correlates with their different profiles of insulitis. As a consequence, we have proposed that these may represent distinct endotypes of the disease; type 1 diabetes endotype 1 (T1DE1) and type 1 diabetes endotype 2 (T1DE2).^40,43^ In this context, the term endotype is used to imply a distinct underlying disease aetiology despite the obvious similarities in clinical outcome.

## Innovations in the analysis of insulitis

The traditional approach used to analyse the cellular composition of pancreatic tissue sections involves the use of immunohistochemical (IHC) methods to stain (and thereby identify) specific cell types with antisera targeted to defined marker proteins. As indicated above, such methods have been extremely useful in revealing the factors associated with disease progression but IHC represents a relatively low-resolution technique which does not easily differentiate between closely related subsets of cells. To address this, newer technologies are now being employed to reveal the deeper complexities of immune cell heterogeneity in insulitic infiltrates and to explore the spatial relationships between the various cells involved. Among these, imaging mass cytometry has been employed in a limited number of type 1 diabetes cases with the advantage that multiple antigens can be targeted in parallel using metal-conjugated antisera.^36,44^ Damond *et al*.^36^ used a panel of 35 such antisera to study 8 pancreases from people with type 1 diabetes covering both recent-onset and longer duration disease. In confirmation of earlier IHC studies,^33^ they observed that both CD8+ and CD4+ are abundant in islet infiltrates and that the numbers of each decline with disease duration. However, in contrast to earlier work pointing to preferential changes in CD8+ T-cell numbers,^33^ the mass cytometry data implicated increasing numbers of both CD8+ and CD4+ T-cell subsets in the islet attack. Despite this difference, the analysis of mass cytometry data confirmed the results of earlier IHC “pseudotime” reconstruction which had revealed that immune cell infiltration is at its height when the estimated rate of β-cell destruction is most intense but then declines as β-cell numbers are reduced.^33^ A key difference from earlier studies was the generalised failure to detect B-cells in pancreatic infiltrates using mass cytometry. A notable exception was one recent-onset case (a child of 13y) who had large numbers of infiltrating B-cells which, as noted above, may be indicative of the existence of disease endotypes.

In a parallel study, Wang *et al*.^44^ also used imaging mass cytometry methodologies to study the proportion of Ki67+ (dividing) lymphocytes in the pancreases of 12 subjects with type 1 diabetes and reported that these were increased among all subsets identified (CD8+, CD4+, CD20+). In particular, they observed that a specific subset of CD4+ T-cells, those with an activated memory phenotype, contained the highest proportion of dividing cells. The functional roles of these proliferating CD4+ cells warrants further attention but the overall state of proliferation among all of the immune cells implies a state of persistent stimulation.

As might be predicted, a large proportion of the CD8+ T-cells infiltrating islets in human type 1 diabetes are reactive against epitopes present in islet proteins, particularly those that are recognised as islet autoantigens.^45^ Among these, pre-proinsulin is a dominant target with between 10% and 40% of infiltrating CD8+ T-cells reactive against this molecule and more than 40 epitopes reported.^45^ Despite this, there is also evidence that some (perhaps many) CD8+ T-cells found in insulitic infiltrates are not reactive against known islet antigens^46^ and the roles (if any) of these cells in disease progression remain to be clarified. It is also uncertain whether the autoreactivity profile of the lymphocytes found in association with islets differs in the two proposed endotypes of type 1 diabetes, although evidence is emerging that this may be the case. Torabi *et al*.^47^ employed Nanostring nCounter technology to interrogate the expression of immune genes in RNA samples extracted from the pancreases of young people assigned to each T1D endotype and identified several genes that were differentially expressed between them. Foremost among these is *IKZF3*, a gene involved in B-cell differentiation which is most abundant in the youngest group of subjects (T1DE1) consistent with the differential involvement of B-cells in the insulitis between the endotypes. Interestingly, and fully consistent with these findings, earlier work had implicated this same gene as predisposing to type 1 diabetes selectively in very young children.^48^ A dozen additional immune genes were also cited as differentially expressed in the pancreas between T1DE1 and T1DE2^47^ suggesting that analysis of such gene expression profiles may provide a fertile means to define the immune cell subtypes present in human islets during insulitis, more fully.

In addition to various classes of lymphocytes, IHC and mass cytometry studies have yielded evidence of the presence of cells of the innate immune system (including mast cells, neutrophils and macrophages) in islet infiltrates.^33,49–51^ Among these, neutrophils have attracted particular attention since their numbers may be depleted in the circulation during the progression of type 1 diabetes and the formation of platelet-neutrophil aggregates (which can promote neutrophil migration) has been reported.^52^ Depletion of circulating neutrophils appears to correlate temporally with the infiltration of these cells into the pancreas in humans and, in at least a proportion of cases, the neutrophils may become activated as judged by the release of neutrophil extracellular traps.^51^ In vitro evidence implies that neutrophils can become activated in response to the induction of endoplasmic reticulum (ER) stress in neighbouring β-cells by virtue of the elaboration of a chemokine, interleukin-8.^53^ It will be important to consider whether release of IL-8 mediates a similar response in islets in human type 1 diabetes but, irrespective of this, increasing evidence implies that cells of the innate immune system should not be overlooked when models of disease progression are built.

As hinted above, we may now be on the threshold of a new dawn in the understanding of human insulitis by the application of high-level, spatially refined, multiplex labelling. For example, an intriguing study has appeared in preliminary form wherein a comprehensive analysis was undertaken in nPOD type 1 diabetes samples, using the Co-Detection by indEXing (“Codex”) multiplex analytical system to target 54 antigens.^54^ The authors concluded that four sub-states of insulitis can be distinguished in human pancreas and that these are differentiated according to the variable status of CD8+ T-cell activation. The presence of an unusual type of immune cell expressing Granzyme B but lacking CD3 was also described. Evidence was further presented that CD8+ cells can accumulate in immature tertiary lymphoid structures located remotely from the islets prior to their migration to target islets; a process which may then involve alterations to the innervation and vasculature of the exocrine pancreas. Tertiary lymphoid organs have also been noted in the pancreas by others^55^ and implicated in mediating immune cell activation.

## Factors driving insulitis

As emphasised earlier (Figure 2), one feature that is common among many type 1 diabetes cases (irrespective of age or immune cell number) is that the infiltrating cells are most frequently arranged around the periphery of inflamed islets. This implies that they access the endocrine compartment from within the exocrine tissue and then migrate towards the islets rather than being extravasated directly from intra-islet capillaries. This would accord with evidence, noted above,^36^ for greater than expected immune cell infiltration within the exocrine tissue of the pancreas in type 1 diabetes and is consistent with the recent proposal that the pancreatic vasculature may be less compartmentalised between islets and exocrine tissue than had long been thought.^56^ The full spectrum of outcomes deriving from this influx remains to be verified but it is noteworthy that the size of the pancreas is markedly reduced in people with type 1 diabetes.^57–59^ This could reflect the loss of one or more factors (not least, insulin) that are trophic for exocrine cells and may also point to a more generalised pancreatic autoimmunity. Alterations in circulating trypsinogen in type 1 diabetes^60^ coupled with changes in other pancreatic enzymes^61,62^ also support this proposition.

If the morphological appearance of 2D sections of pancreas provides a true reflection of the primary route of access by which immune cells reach islets, then they will inevitably encounter a further barrier before gaining access to the β-cells. This is because each islet is surrounded by a capsule comprised of collagen, laminin, and other structural proteins as well as a basement membrane.^63^ Thus, immune cells must penetrate this physical barrier in order to reach the β-cells and mathematical modelling implies that this is likely to be a rate-limiting step.^64^ Very little is known about the mechanisms involved in breaching the barrier but it is probable that proteolytic enzymes are involved and there is physical evidence that the capsule loses integrity at sites where immune cells are localised.^65^ Identification and targeting of the enzymes involved might provide a means to delay β-cell demise therapeutically, if this goal could be achieved with a high degree of selectivity.

In the foregoing discussion, the tacit assumption has been made that the lymphocytes targeting β-cells are attracted by virtue of the breakdown of immune tolerance. This, in turn, implies a measure of recognition between the two which culminates in immune-mediated β-cell loss. This may operate at two levels. Firstly, there is likely to be a chemotactic gradient emanating from the islets which serves to direct autoreactive lymphocytes to their target β-cells. The precise identity of the chemoattractant(s) is unclear although increases in chemokines such as CXCL10 have been reported in islets in type 1 diabetes.^66^

**Figure 3.**
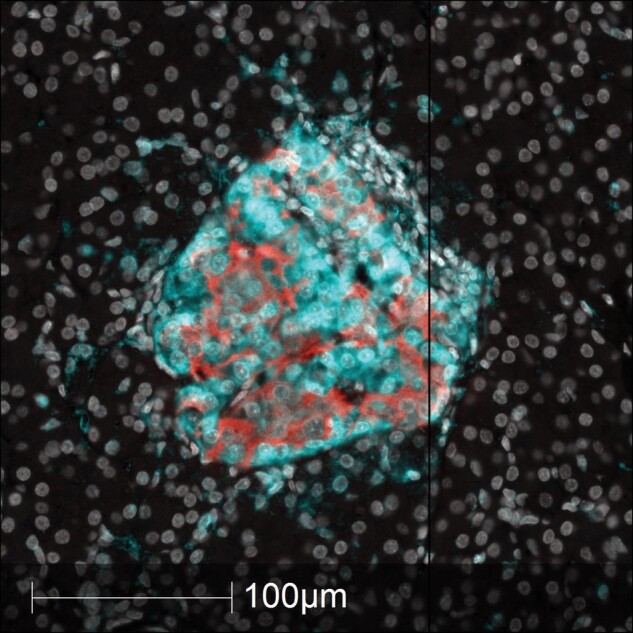
Hyper-expression of class I MHC antigens on the cells of an inflamed islet of Langerhans from a subject with recent-onset type 1 diabetes. High power image of a single islet of Langerhans from a subject with type 1 diabetes immunostained to reveal the expression of insulin (red) and class I MHC (cyan). The islet has a peripheral contingent of lymphocytes to the upper right (distinguished by small, intensely white nuclei). As is typical of insulin-containing islets in type 1 diabetes, all of the endocrine cells hyper-express class I MHC molecules. Certain cells (including some immune cells) extending beyond the islet boundary are also stained intensely (cyan) suggesting elevated levels of class I MHC.

A second mechanism of target recognition by immune cells is via the display of antigenic peptides on the surface of β-cells by major histocompatibility antigens (MHC) molecules. In particular, CD8+ T-cells are induced to kill target cells by mechanisms involving recognition of relevant (“non-self”) peptides which are processed and displayed on MHC class I molecules.^67,68^ Importantly, a hallmark feature of type 1 diabetes is the hyper-expression of MHC-I on islet cells which are induced to reach levels well beyond those found in the islets of control subjects without diabetes.^69^ Elution of the peptides that are displayed on islet cell MHC-I under these conditions suggest that these could be involved in immune cell targeting, especially as they are likely to become displayed at increased intensity as a result of the MHC-I hyper-expression. However, this mechanism relies on the ability of influent CD8+ T-cells to directly access the displayed peptides by physical contact and, as explained above, direct cell–cell contact may occur to only a limited extent during the progression of human type 1 diabetes. There is, in addition, a further possibility that combines both of these concepts since it is known that MHC-I molecules can be exported from cells under certain conditions, including type 1 diabetes.^70^ Our own unpublished data imply that β-cells retain this capacity and it is conceivable (but not proven) that MHC-I molecules bearing antigenic peptides might be secreted from β-cells during type 1 diabetes thereby generating a chemoattractant gradient to autoreactive CD8+ T-cells. In this situation, the β-cells could be considered to be actively complicit in mediating their own demise by acting to promote insulitis (Figure 3).

While β-cells may be at least partly involved in driving autoimmunity, it is also clear that, once the process has begun, they do not entirely assume the role of passive victims awaiting an inevitable demise. Rather, they can establish robust mechanisms to mitigate the autoimmune attack.^71^ Somewhat counterintuitively, these also include the hyper-expression of MHC-I since β-cells express not only classical molecules involved in CD8+ T-cell activation but they also upregulate “non-classical” MHC-I molecules which can resist immune-mediated attack.^71^ Molecules such as MHC-E, F, and G become hyper-expressed on β-cells in type 1 diabetes and can engage influent innate immune cells to down-regulate their cytotoxicity. In addition, one of the β-cell responses to cytokine (notably interferon-α) exposure is induction of PDL-1,^72^ an immune checkpoint protein which can bind to its ligand, PD-1, displayed on CD8+ T-cells to initiate T-cell death. Furthermore, β-cells also express a second immune checkpoint protein, CD47,^73,74^ which can both deflect attacks by incoming macrophages and also actively promote their own (or neighbouring β-cell) viability via interaction with signal regulatory protein-α (SIRPα). There has been debate about whether β-cells display SIRPα under normal conditions^73,74^ but, despite the apparently limited mRNA expression, functional and histological evidence suggest that SIRPα is present at the protein level (and is induced by cytoprotective agents such as interleukin-13^75^) such that it may also be involved in repelling autoimmunity.

## Conclusions

Overall, it is evident that many questions remain about the role and mechanisms by which insulitis leads to β-cell demise in human type 1 diabetes. Indeed, one key question that remains unanswered is whether progression to type 1 diabetes reflects the early death of β-cells mediated by influent lymphocytes. Importantly, the answer may be different in young children (<7y) vs those who are older (>13y) at onset (where β-cell loss is less profound). A second outstanding issue is the site at which the disease is initiated; whether at the level of the immune system or in the β-cells. The answers to both questions are important but, irrespective of this, it is clear from the positive therapeutic outcomes heralded among subjects receiving the anti-CD3 monoclonal antibody, teplizumab,^76^ that activated lymphocytes ultimately play a key role in the disease. Nevertheless, additional painstaking work is still required to further penetrate the shroud that continues to envelop the enigmatic process of insulitis. This effort can be expected to pay additional dividends by pointing the way to ever more targeted means to intervene successfully in disease progression.

## Data Availability

Relevant images from the Exeter Archival Diabetes Biobank are available from the author on reasonable request and many can be viewed online at: https://www.pancreatlas.org
